# An Inaugural Dissertation on Dental Decay

**Published:** 1850-04

**Authors:** J. W. Keyes

**Affiliations:** Florida


### AN INAUGURAL DISSERTATION ON DENTAL DECAY;

SUBMITTED TO THE FACULTY OF THE OHIO COLLEGE OF DENTAL SURGERY, FOR THE DEGREE OF DOCTOR OF DENTAL SURGERY.

By J. W. Keyes—*Of Florida.*

"The human frame, offspring of Heaven's high will,
Displays throughout inimitable skill;
No part defective: none that perfect love
Could prompt unbounded wisdom to improve."

Dentologia, page xxxiv.

Beauty, order, and perfect adaptation, mark all the works of nature; and whether we consider the elements, note the individual parts, or contemplate the harmonious whole, we are filled with a sense of the infiniteness, the unbounded wisdom and goodness of the Arch-architect who constructed them. Nor do we find less food for study, or less wisdom to admire, when we contemplate the effects of a violation of the laws which direct, govern, and control the functions of all organized matter. And nowhere do we find these truths more clearly and forcibly illustrated than in the study of the construction, functions, and diseases of the teeth. Man being, unlike any other animal, an inhabitant of every portion of the earth, from the bleak and frigid regions of the north, to the burning zone of the equator, and feeding on every variety of food, from grass to fruits, and from fish to coarsest flesh, has these organs admirably arranged to suit his varied and ever-varying habits. Thus the broad, flat, and sharp, are for cutting; the long, thick, strong, conical, for tearing; the double, cutting-edged, for hashing; and lastly, the broad, uneven, for grinding. As they are exposed to frequent and great variations of temperature, to acids, and to sweets, and required to masticate hard, resisting food, and incapable, to any great degree, of rebuilding their structure, they are endowed with but little sensibility, and composed of hard, unyielding material, which is not readily worn and wasted away by use. And they are not so connected with the system as to render the removal of one, a material loss, or the retention of all, a necessary condition of life. They are continually bathed in thick, and somewhat viscial fluids, calculated to protect them greatly from the injurious action of many hurtful agents, and the ever-busy tongue is in their midst, thrusting over, around, and between, to remove any foreign matter which may be lodged among them.

When these facts and circumstances are considered, we are brought to the conclusion that these organs must be exempt from disease; but almost every mouth bears indubitable evidence to the contrary; and an examination of the subject shows them to be *more* frequently diseased than any other organs in the body. To inquire briefly into the *cause* of one of the diseases, is my object.

We find decay of the teeth mentioned by some of the earliest writers on medicine; but its causes do not appear to have received much attention until within comparatively a late period. Indeed, it is only since dentistry began to be practiced as a distinct branch of medicine, that this subject has elicited, except to a very limited degree, that attention which its great importance demands. In the medical and dental professions, great diversity of opinion has prevailed as to the *cause* of dental decay, and it seems, from an examination of some of these opinions, that they are the result of careless, rather than close and constant examination. In some instances, indeed, we are led to believe that no attention had been given to the subject, except so far as to enable the writer to form a *theory*. The importance of the subject, though by far too lightly estimated, was such that writers on medicine could not pass it over in silence; but still it seems not to have received that study and laborious research which was bestowed upon many minor, and oftentimes even insignificant subjects. Thus we have Ambrose Pare affirming that decay is caused "by inflammation," and John Hunter, one of the brightest lights of the medical profession, publishing as his opinion, that "caries is a disease arising originally within the tooth itself." Ruspini thought it proceeded from "internal and external causes," whilst Gerbeaux held that it originated in a "transmissible organic disposition." Fox taught that the proximate cause was "inflammation in the bone of the crown of the tooth, and that the chief predisposition consisted in a defective formation either of the bone or enamel;" Hertz, that "heat and increased circulation in the gums were the causes of decay;" Berr, that it was the result of "lateral pressure of the teeth against each other;" Bell, that it was produced by "inflammation;" Saunders thinks it results from interruptions and commotions in the system, occurring during their formation, and violent and unnatural actions and uses, such as cracking nuts, biting hard substances, &c., &c.; Liston gives as one of the causes "an unhealthy state of the system;" and lastly, in this day, when the labors of surgeon dentists have thrown so much light on this subject, and every teacher of medicine should be acquainted with the progress of the science in all its ramifications, but especially upon a subject claiming so much of public and professional attention, we find our distinguished surgeon and pathologist, Prof. Gross, advancing the exploded doctrine, that "caries of the teeth is strictly analogous to necrosis of the bone."

Whilst so many able and active members of the medical and dental profession, whose pursuits led them directly to this important subject, have so *Grossly* neglected it, there are others who have diligently, faithfully, and perseveringly studied it, and by their labors have rendered themselves in an eminent degree the benefactors of mankind.

It is by many contended that decay *always* commences *internally*, and among those who entertain this opinion are

Fox, Bell, Gross, and others. By some it is believed that it commences both *externally* and internally, and among these are Hunter, Ruspini, &c. And by others again, and by far the greater number too of those who have devoted much time and patient study to the subject, it is contended that it *always* commences externally, and among these, of our own country, we may mention Parmley, Brown, Harris, Taylor.

The causes of dental decay, I think may most properly be divided into *direct* and *indirect*, and the *direct* causes may be briefly stated to be ACIDS. By referring to the record of opinions upon decay of the teeth, we find that at an early day the action of acids was set forth as a cause. The "corrosive action of acids" was published in London by Salmon, in 1664; but to Eleazar Parmley is ascribed the honor of having first taught that doctrine in this country. At this time, I believe, a majority of the most scientific dentists acknowledge acids to be, at least, the principal agent in the destruction of the teeth.

Prof. Harris, of Baltimore, in his work on dental surgery, remarks that he is "aware that according to the tables of elective *attractions*, oxalic, tartaric, sulphuric, and succinic acids, are the only ones which have a greater atraction for lime than *phosphoric;*" but he does not seem to be aware that tables of electric attraction have long since become an obsolete idea; for he says "it may hence be argued that no other acids are capable of decomposing or prejudicially affecting the teeth." Since the action and reaction of elements and compounds is so modified and controlled by circumstances, it is clear that tables of electrive attraction upon which reliance could be placed, cannot be made. The fallacy of those to be found in the older chemistries is amply pıoved by a series of useful and entertaining experiments, made by Prof. Wescott, of Baltimore, and detailed in an article published in the American Journal of Dental Science. Teeth were submitted to the action of citric and acetic acids, and the enamel was so corroded in forty-eight hours, that much of it was easily removed with the finger nail. Muriatic and nitric acids, though largely diluted, soon decomposed the teeth, and raisins so corroded the enamel in twenty-four hours, that it presented the appearance, and was of the consistence of chalk. Oxalic, muriatic, nitrous, nitric, and lactic acids, have been found generated in the mouth, and acetic acid, it is well known, is constantly produced by the fermentation of particles of food lodged in the depressions and between the approximal surfaces of the teeth, besides being in constant and very general use as a condiment." Often, from imprudence in diet and in some diseases, we have acid ejected from the stomach, so acrid and corrosive as to put the teeth "*on edge.*" In many diseases there is a vitiated condition of the secretions, rendering them acid, and favoring the formation of acid in the mouth. Mallic, citric, tartaric, lactic, muriatic, sulphuric, and nitric acids, are taken with our food and drink and as medicines. Fruits being and by lodging some of their particles between, retain their acids in constant and immediate contact with the teeth. And it is, I am inclined to believe, by the decomposition of a portion of the enamel, and the removal of the mucous coating, and rendering it rough and uneven, that the disagreeable sensation designated as the teeth "*on edge,*" is produced from eating sour fruits, &c., &c.

Periera recommends, as a dentifrice, the bitartrate of potash; and one of our distinguished authors advise the use of dilute sulphuric acid for the removal of salivary calculus; and these articles have been extensively used.

Thus we see that, either alone or in combination, a great variety of acids, capable of decomposing the teeth, are brought in contact with them, and the wonder would seem to be, not why so many teeth decay, but *how so many* remain healthy.

We must, however, bear in mind, that the teeth are not so easily acted upon in the mouth as out of it; for the ris conservatrix naturæ, the mucus and saliva, tend to protect these secretions, which are alkaline, and in which they are bathed, neutralize to some extent, and dilute to a great degree the acids, whilst the mucus, by its viscid and adherent properties, forms a protecting coat over them. And here, en passant, we notice that infinite wisdom and foresight which is everywhere manifest in the works of nature, adapting everything so perfectly to the purpose for which it is intended, and throwing agents around, about, and within, to shield us from the numberless ills which beset us.

The salivary glands are so endowed that they pour out their fluid in greatly increased quantity, when an acid is taken into the mouth—indeed, even at the thought of anything sour, the mouth is flooded with this secretion.

It may be well to notice, in connection with this part of my subject, that Andral's opinion, from some experiments, is, that the "mucus is *acid*." "The mucus examined was taken from the mouth in the morning, before food was taken or any considerable quantity of saliva secreted." But since it has been shown, that *mucin*, which is the chief organic constituent, is an albuminous compound held in solution by an alkali, and mucus, by most physiologists, is regarded as alkaline, or, at most, neutral, may we not conclude the acid reaction in M. Andral's experiments, was owing to an acid generated *after* the mucus was secreted? It seems to me *highly probable*.

Robinson, in his work on the teeth, says: "The labors of recent pathologists seem to show that an acid liquor is secreted by the glands of the gums contiguous to the teeth, and that it is to the agency of this liquor that caries is contingently due." This *discovery* seems to me so utterly repugnant to common sense, and is so much at variance with my notions of the harmonious perfection of nature's works, that I at once reject it. I cannot believe that nature would construct glands, the healthy function of which should be to secrete a fluid which would so probably destroy the organs she has so wonderfully constructed, and upon the perfection of which so much of the health and happiness of her creatures depend.

We come now to the *indirect* causes. A majority of the writers upon dental decay seem to have confined their researches to the predisposing or *indirect* causes, and hence, among *direct* causes, we have enumerated "organic disposition," "defective formation," "heat," "cold," "increased vascularity of the gums," "lateral pressure against each other," "uncleanliness," &c., &c., &c. All these should be classed among the *indirect* causes, and these we may add "everything productive of irritation in the alveolar dental membranes and gums," and every disease or condition of the system vitiating, acidifying, or decreasing the secretions around these organs. There is an action and reaction, a cause producing its effect and the effect becoming a cause, to a very great extent in affections bearing upon the teeth. Thus defective formation, uncleanliness, &c., &c., produce impaired digestion and vitiation of the secretions, which, acting in their turn, increase that condition most favorable to the destruction of these organs. In dyspepsia, we have that acid state of the fluids which corrodes and wears away the teeth, and then imperfect mastication and the ingestation of the viscious and poisonous products of the diseased denture, gives rise to a continuous dyspepsia, with its attendant ills. Instances might be multiplied, but these may suffice. Pressure, blows, fractures of the enamel, irregularity, crowded condition, deposit of tartar, recession of gum, and indeed everything which exposes the bone or dentine, generates or favors the retention of an acid among the teeth, may be regarded as an *indirect* cause. I suppose one great reason why acids act more rapidly upon the bone and dentine than upon the enamel, is because of their greater porosity. The acid is rapidly absorbed, and is brought into intimate contact with a much larger surface, it is, as it were, applied to all sides of every *atom*. According to this view we may account for the greater liability and more rapid decay of the teeth prior to maturity than after. In the young subject they are—comparatively—highly vital, very porous and vascular, but as age advances, they become gradually more and more solid, more and more lifeless, and less liable to decay, and especially that rapid destruction which may occur in early life.

I must here add, what might perhaps with more propriety have been said in another place, that the modifications which decay presents, is ascribable to the presence, absence, or degree of inflammation in the dentine.

Taking the view of dental decay which I have, the remedies very naturally suggest themselves, and as "prevention is better than cure," it should be one of our duties to enforce, by precept and certainly by example, that best and surest of all preventive measures—*perfect cleanliness.*

I might here very properly and by a most natural transition, pass on to notice the *consequences* of dental decay. A wide field here opens before me, and I might swell this dissertation into a volume. I might trace the various influences which diseased teeth exert upon the nervous system generally—causing, continuing, aggravating and complicating the diseases to which it is liable. I might notice its effects also, in diseases of the digestive apparatus; and from these to all organs as being associated. I might turn to watch its local diseases, such as alveolar abscess, which may become a very serious disease, by the matter accumulating beneath the superficial facia of the neck—forming a large unsightly tumor, or it may burrow beneath the deep seated facia and empty into the thorax, &c. &c.—or disease of the antrum, epulis, ulceration of the throat, lips, &c.—tonsilitis, neuralgia in its many and distressing forms—azaena, spina ventosa of the jaw, and osteo-sarcoma may supervene upon this last disease," and very many others might be enumerated—and their rise, progress and treatment, together with *their* terminations and consequences—considered—but I have already written more than the merit of the writing warrants.

